# Experimental comparison between Nb_2_O_5_- and TiO_2_-based photoconductive and photogating GFET UV detector

**DOI:** 10.1038/s41598-023-34295-5

**Published:** 2023-05-02

**Authors:** Zahra Sadeghi Neisiani, Mahdi Khaje, Abdollah Eslami Majd

**Affiliations:** Malek Ashtar University of Technology, Tehran, Iran

**Keywords:** Biochemistry, Biophysics, Biotechnology, Health care, Health occupations, Medical research, Engineering, Materials science, Nanoscience and technology, Optics and photonics, Physics

## Abstract

In the present study, by adding graphene to a photoconductive photodetector with a niobium pentoxide (Nb_2_O_5_) absorber layer and exploiting the photogating effect, the responsivity of the photodetector is significantly improved. In this photodetector, the Nb_2_O_5_ layer detects the light, and the graphene improves the responsivity based on the photogating effect. The photocurrent and the percentage ratio of the photocurrent to dark current of the Nb_2_O_5_ photogating photodetector are compared with those of the corresponding photoconductive photodetector. Also, the Nb_2_O_5_ photoconductive and photogating photodetectors are compared with titanium dioxide (TiO_2_) photoconductive and photogating photodetectors in terms of responsivity at different applied (drain-source) voltages and gate voltages. The results show that the Nb_2_O_5_ photodetectors have better figures of merit (FOMs) in comparison with the TiO_2_ ones.

## Introduction

Two-dimensional materials can interact strongly with light. One of these two-dimensional materials is graphene, which is a monolayer of carbon atoms tightly arranged in a crystal network with a two-dimensional honeycomb pattern^1^. The first synthetic single-layer graphene was made and characterized by Game and Novoselov in 2004. For this invention, Game and Novoselov received the Nobel Prize in Physics in 2010^2^. In 2013, two-dimensional atomic sheets of graphene and a new classification of nanoscale materials that can be used in electronics were proposed^3^. The exciting properties of graphene include high charge mobility, excellent thermal conductivity, and high strength^1,4–7^. One of the applications of graphene is in photodetectors. Single-layer graphene can absorb only 2.3% of the irradiated light from 300 to 2500 nm, so it has low responsivity^8^. The responsivity of graphene photodetectors can be improved in different ways, including the use of microcavity structures^9^, quantum dots^10^, graphene nano disc arrays^11^, graphene waveguides^3,12,13^, heterostructures, and graphene incorporated with different materials^14,15^. High responsivity was reported in the graphene photodetectors with photogating effect^16–18^. In 2010, a graphene detector was made. The graphene detector had a responsivity of 6.1 mA/W at a wavelength of 1.55 μm^19^. In 2012, a graphene detector with a cavity was presented. The detector had a responsivity of 21 mA/W at a wavelength of 850 nm^9^. In 2015, a graphene and boron nitrate detector with an optical waveguide was presented; this detector had a responsivity of 0.36 A/W^20^. Various studies have been conducted on graphene and light absorption processes, including photovoltaic effect (generating photocurrent based on electron–hole separation under the electric field at the junction of the regions with different impurities), photogating effect (light absorption that changes the density of the carriers, which in turn leads to a change in the conductivity of the component in the transistor structure), bolometric effect (change in conductivity as a result of light radiation and temperature increase), and thermoelectric effect (creating voltage according to the Seebeck effect due to the increase in the temperature of carriers)^21^. Since the responsivity of the photogating effect is much higher than that of other effects, the present study focuses on this effect. In photogating effect, one of the carriers is trapped in the absorber layer. In other words, the lifetime of the additional carriers increases as the carriers are separated from each other by defects and impurities. If one type of the generated carriers is trapped, it can generate an additional electric field such as a gate voltage to modulate the channel conductance^22–26^. Such detectors with small dimensions show high responsivity and limited response speed due to the prolongation of the lifetime of the additional carriers^27^. In 2012, a graphene structure with quantum dots was proposed. In this structure, a responsivity of 10^7^ A/W was obtained at a wavelength of 532 nm^10^. In 2016, graphene∕SiO_2_/lightly-doped Si structure was used for high sensitivity and responsivity. The operating range was from the visible to near-infrared regions, and the responsivity was 1000 A/W at a wavelength of 514 nm. In this photodetector, due to the defects between SiO_2_ and the lightly-doped Si, electrons accumulate in the traps and create a negative gate voltage, causing more holes to be induced and thus increasing a high gain^25^. In 2018, black phosphorus (BP) with a direct bandgap of 0.3 eV was used as a light-absorbing material. At wavelengths of 655 nm, 785 nm, and 980 nm, the responsivities of 55.75 A/W, 1.82 A/W, and 0.66 A/W were obtained, respectively. Excited electrons are trapped in trap levels, and the holes pass through the graphene layer by the internal potential between graphene and BP. The lifetime of carriers increases with traps. Due to the high mobility of graphene, holes can flow in the circuit before recombining with electrons. The introduced structure works in the visible to near-infrared regions based on the photogating effect^22^. In 2018, Ti_2_O_3_ nanoparticles with a band gap of 0.09 eV were used to fabricate a detector in the mid-infrared spectrum. The mechanism is the same as before. This detector had a responsivity of 300 A/W for a wavelength of 10 μm^28^. In 2018, the photogating effect in graphene photodetectors was investigated using SiO_2_/n-doped Si substrate. At wavelengths of 450 nm and 1064 nm, the responsivities were 500 A/W and 4 A/W, respectively. The bending of the band at the Si/Si*O*_2_ interface separates the electron–hole pairs. Under the electric field, the electrons move towards Si while the holes are trapped at the Si/SiO_2_ interface; the accumulation of holes at the Si/Si_2_ interface acts like a positive gate and increases the Fermi level of graphene. This causes the graphene to become n-type^24^. In 2018, a graphene transistor was fabricated with an indium antimonide (InSb) substrate. A responsivity of 33.8 A/W with a photogating effect at a wavelength of 4.6 μm was achieved^29^. In recent years various photodetectors with TiO_2_ and Nb_2_O_5_ absorber layers in the UVA region have been presented^30^. In 2011, an Nb_2_O_5_ nanobelt was proposed, and at 1 V, a responsivity of 15.2 A/W was obtained^31^. In 2015, an Nb_2_O_5_ nanoplate photodetector was fabricated with a responsivity of 24.7 A/W at 1 V^32^. In 2021, a MAPbI_3_ nanowire photodetector was fabricated, and a responsivity of 20.56 A/W at 1 V was reported^33^. In 2023, a type-II heterojunction of TiO_2_ NTs/Cs_3_Cu_2_I_5_ nanoparticles hybrid fiber was presented with a responsivity of 26.9 mA/W at − 1 V^34^. The photogating effect can be investigated in three different structures, namely quantum dot^10^, bulk^23–25^, and thin film structures^8^. Quantum dots can be integrated into two-dimensional materials to achieve some advantages. As the first advantage, quantum dots with greater thickness resolve the issue of the low optical absorption of two-dimensional materials. The second advantage is that two-dimensional materials have high carrier mobility, and the third advantage is that some two-dimensional materials do not have a wide absorption spectrum while quantum dots compensate for this limited responsivity. For a two-dimensional material such as graphene, there is no mechanism to produce multiple carriers from one photon. By using quantum dots, a large number of holes can flow in the circuit, and as a result, the gain increases. This is because the lifetime of the trapped electrons is long, and the mobility of carriers in graphene is high. One of the disadvantages of quantum dots is their toxicity. Moreover, the dimensions of quantum dots change the bandwidth of the used materials. In bulk detectors, due to the defects between SiO_2_ and lightly-doped Si, electrons accumulate in the traps and create a negative gate voltage, causing the induction of more holes and thus increasing the gain. In other words, the bending of the band at the Si/SiO_2_ interface separates the electron–hole pairs. Under the internal field, the electrons move towards the Si substrate while the holes are trapped at the Si/SiO_2_ interface, and the accumulation of holes at the Si/SiO_2_ interface acts like a positive gate and increases the Fermi level of graphene. As a result, the graphene is converted into n-type graphene. A highly-doped silicon substrate is not used since it has additional carriers with a much shorter lifetime. The application of the bulk structure is limited to high-energy materials and X-rays^23–25^.

In the present study, Nb_2_O_5_ (3.7 eV) and TiO_2_ (3.2 eV) absorber thin films are used based on the photogating mechanism. The use of broadband materials is an advantage for the photodetector because it operates at room temperature. By transferring graphene to the photoconductive detector, the responsivity increases approximately 20 times. The use of an Nb_2_O_5_ absorber layer is a new technique. The advantages of this technique are its low cost and simplicity of obtaining Nb_2_O_5_ without any special technology only by combining a few solutions. In the present study, a photoconductive detector with an Nb_2_O_5_ absorber layer and a graphene photogating photodetector with the same Nb_2_O_5_ absorber layer are compared in terms of responsivity and the percentage ratio of photocurrent to dark current. The responsivities of the photogating and photoconductive detectors with the Nb_2_O_5_ absorber layer are 12.69 A/W and 0.65 A/W respectively. The percentage ratios of photocurrent to dark current of the photogating and photoconductive detectors with an Nb_2_O_5_ absorber layer are respectively 2.84% and 0.16% at a drain-source voltage of 1.5 V and a gate voltage of 1 V. The responsivities of the TiO_2_-based photoconductive and photogating detectors, which are fabricated under the same laboratory conditions, are 0.45 A/W and 8.32 A/W, respectively. The percentage ratios of the photocurrent to dark current of the TiO_2_ photoconductive and photogating detectors are 0.16% and 2.84%, respectively. The responsivity of a photogating detector with an Nb_2_O_5_ absorber layer is about two times higher than that of a TiO_2_ photogating detector.

## Fabrication process

**Figure 1:**
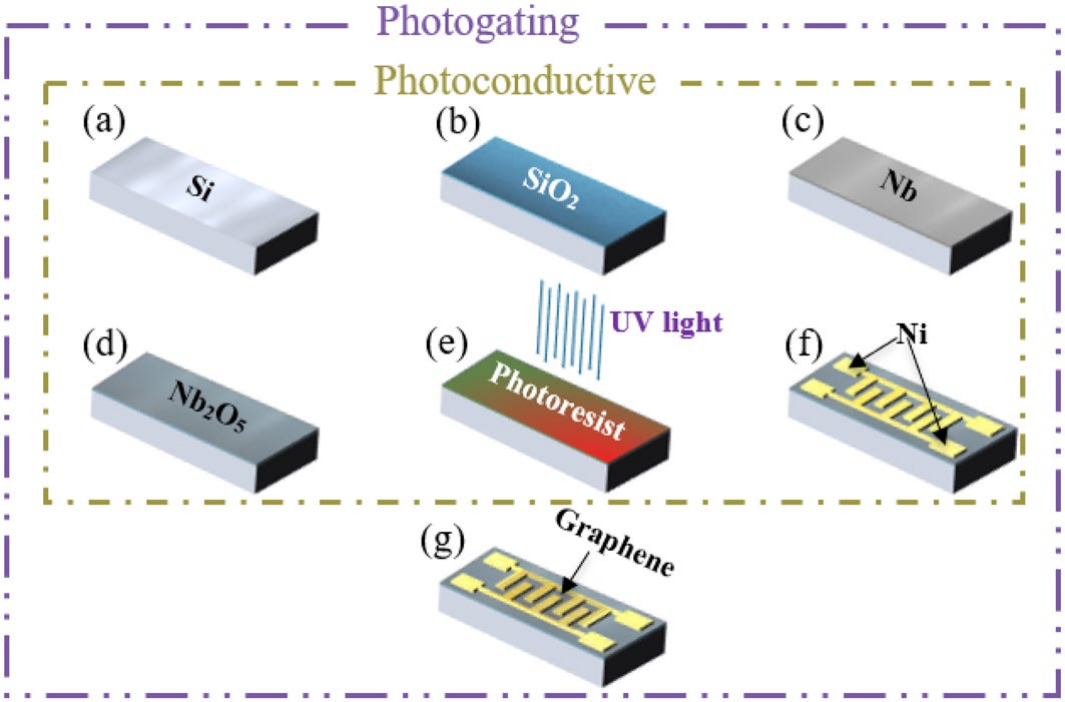
(**a–f**) Photoconductive photodetector fabrication steps with an Nb_2_O_5_ absorber layer and (**a–g**) photogating photodetector fabrication steps with an Nb_2_O_5_ absorber layer and graphene.

The fabrication steps of the photoconductive and photogating photodetectors with an Nb_2_O_5_ absorber layer are shown in Fig. 1a–g, respectively. Figure 1a shows a p-type silicon wafer with a thickness of 430 μm along the (100) direction. The silicon surface was cleaned by the RCA method. As shown in Fig. 1b, an oxide layer was formed on silicon with 300 nm thickness using thermal oxidation. The doping amount was 11–13 Ω/cm, and the leakage current density of the oxide sample was 0.205 A/m^2^. As shown in Fig. 1c, 30 nm Nb was deposited on the SiO_2_ layer by electron-beam physical vapor deposition. Then, as shown in Fig. 1d, an anodic process was used to form an 81-nm Nb_2_O_5_ absorber layer on SiO_2_. For the anodization of Nb, an electrolyte consisting of 1200 ml of ethylene glycol C_2_H_6_O_2_, 800 ml of H_2_O, and 160 g of (NH_4_) B_5_O_6_ was used. The Nb layer was connected to the positive pole and the platinum electrode, which was inside the anodization solution, was connected to the negative pole, and the anodization process was carried out. As shown in Fig. 1e,f, the interdigit electrodes were patterned on the structure using the lithography process. For this purpose, a glass mask was used. The width of each metal electrode was 10 μm, and the width of the transistor (w) was 5000 μm. The distance between two metal electrodes (length) was 12.5 μm, and the number of the distances (n) was 249. Accordingly, as shown in Eq. (1), the total width of the transistor is 1245 mm.

1 $$W_{Total} = n \times w,$$

where W_Total_ is the total width of the transistor, n is the number of distances, and w is the width of the transistor.

To fabricate the photogating photodetector, all the steps are the same as performed for the photoconductive photodetector; only at the end, as shown in Fig. 1g, graphene is transferred to the final photoconductive structure. In this structure, graphene, which was the product of the Graphene Company and grown using the chemical vapor deposition (CVD) method, was used^35,36^.

**Figure 2:**
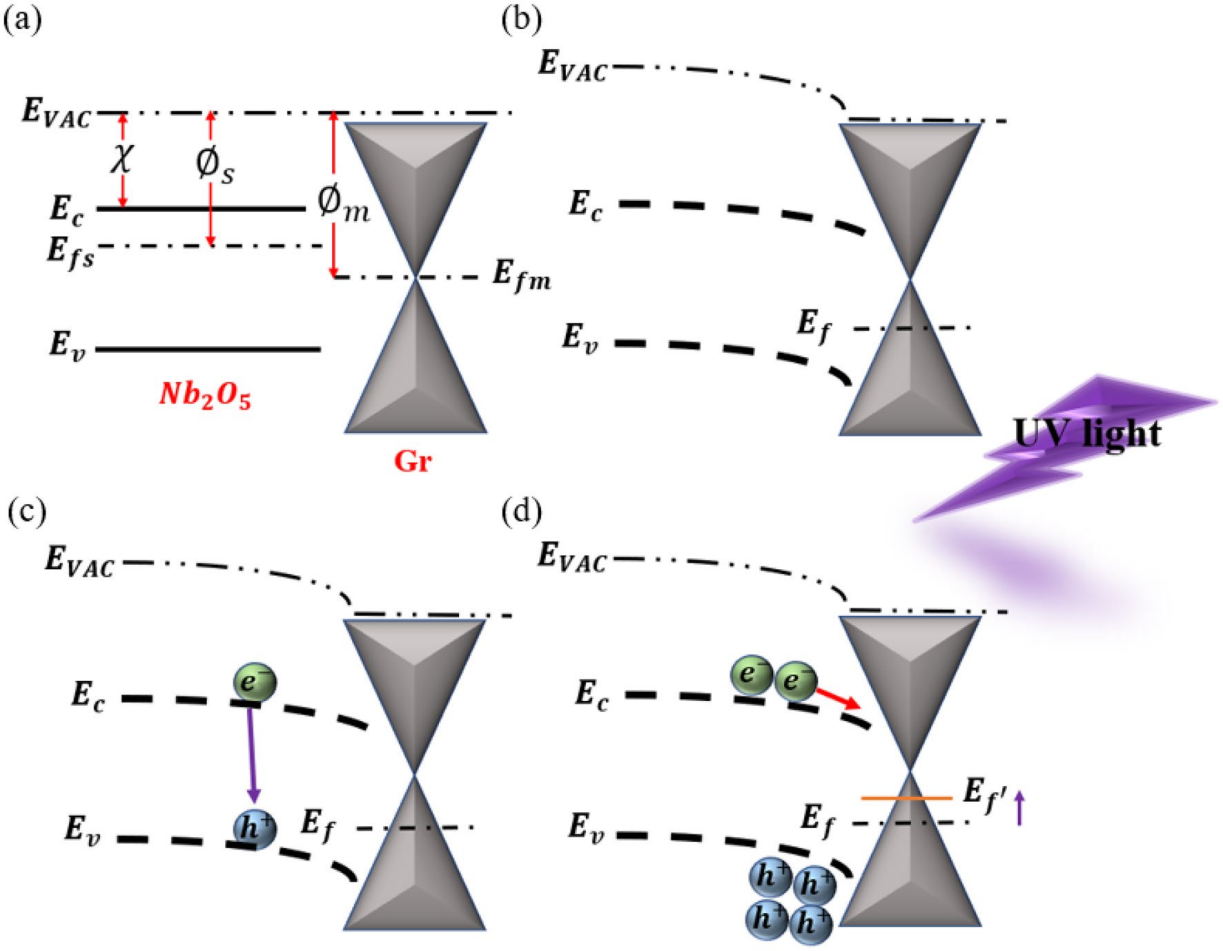
(**a**) energy level of graphene and Nb_2_O_5_, (**b**) energy level of graphene and Nb_2_O_5_ when they are bound to each other, (**c**) UV light radiation and electron–hole pair production in the Nb_2_O_5_ layer, and (**d**) accumulation of holes behind the built-in potential barrier at the graphene/Nb_2_O_5_ interface and the change in the Fermi energy level of graphene^37^.

Figure 2 shows the energy bands for graphene and Nb_2_O_5_ heterostructures. By placing the graphene monolayer on the Nb_2_O_5_ layer, due to the difference in the Fermi level of the two materials, a built-in potential barrier is created between the two materials. Then, light radiation in the range of the band gap of the Nb_2_O_5_ layer leads to electron–hole pair production inside the Nb_2_O_5_ layer. Due to the potential barrier between the graphene and Nb_2_O_5_ layer, the electrons move towards the graphene layer, but the holes are trapped inside the Nb_2_O_5_ layer. The trapping of the holes changes the Fermi energy level of graphene and the resistance of the graphene channel, causing a large photocurrent^22–26^.

Graphene was transferred through a wet transfer method in seven stages, including etching the copper layer, substrate preparation, and graphene transferring. The process begins by eliminating the unwanted graphene that is placed under the copper layer during the CVD process. This process was done in a 20% nitric acid solution in which the substrate was kept for about five minutes. Then, the copper was etched using 0.2 M iron (III) chloride for almost two hours. After the metal residues were removed by RCA, the substrate was ready for graphene transferring, but before that, the substrate must be prepared. Next, the substrate was immersed in piranha solution at 70 $^\circ{\rm C}$ for about 15 min. Then, graphene was transferred to the prepared substrate. The following two stages were performed to increase the adhesion at the graphene/substrate interface and remove poly(methyl methacrylate) (PMMA). The substrate was heated moderately using a heater to a temperature of 80 $^\circ{\rm C}$ for about five minutes before being subjected to a higher temperature of 130 $^\circ{\rm C}$ for approximately 20 min. Next, the substrate was allowed to cool down for a few minutes at room temperature. The process finished by removing PMMA via an *N*-Methyl-2-pyrrolidone (NMP) solution at a temperature of 70 $^\circ{\rm C}$ for about 15 min.

**Figure 3:**
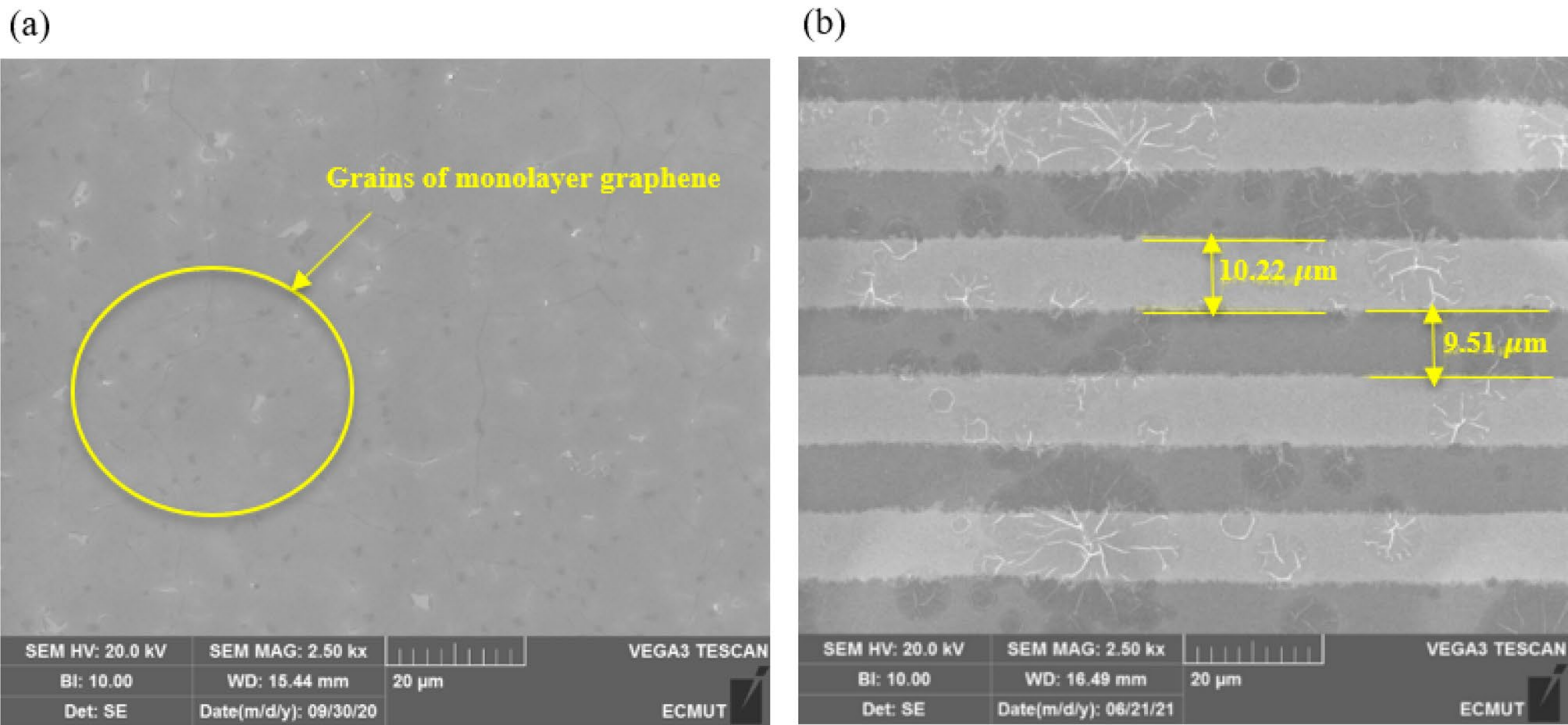
(**a**) Grains of monolayer graphene and (**b**) a sample of interdigit electrode design on the Nb_2_O_5_ absorber layer.

Figure 3 shows the scanning electron microscopy (SEM, Tescan VEGA3) images of the graphene transferred onto the SiO_2_ layer and interdigit electrodes to the Nb_2_O_5_ layer. Figure 3a shows the grains of the monolayer graphene that was annealed for one hour at 550 ℃ and a vacuum of 4.4 $\times$ 10^–6^ Torr to remove the PMMA residues. The average graphene sheet resistance was obtained by the van der Pauw method. An HP4450 semiconductor measurement device was used at a voltage of 1 V and a current of 1 μ$\mathrm{A}$. The sheet resistance of the graphene layer was around 1600 $\pm 10\%$ ohms/square. An example of the interdigit electrode design is shown in Fig. 3b. The contact resistance of graphene and nickel electrodes were measured to be about 640 Ω μm ± 15% using the transmission line method (TLM). Fortunately, the contact resistance was good enough such that it did not affect the process of fabricating the photodetector although a lower contact resistance would be better.

## Equations

In the photogating effect, if one species of the generated carriers is trapped, it can generate an additional electric field, in the form of the gate voltage, to modulate the channel conductance. Photodetectors with small dimensions show high responsivity. In the photovoltaic effect, the produced electron–hole pairs are recombined in picoseconds. However, the carrier lifetime in the photogating effect is longer than that in the photovoltaic effect, i.e., 1 s compared to around 20 ms^27^. The gain can be obtained using Eq. (2). As can be concluded from this equation, responsivity and photocurrent increase with the increase in the trapping time and the decrease in the time interval during which carriers pass through the graphene channel (transit time). As the graphene channel becomes shorter, the photocurrent and responsivity increase, while the effective area of the photodetector decreases^38^.

2 $$G = \frac{{\tau_{life} }}{{\tau_{tr} }}.$$

The time during which free charge carriers travel from drain to source is denoted by “τ_tr_” and the time interval during which these free charge carriers are trapped near nanoparticles is denoted by “τ_life_”.

If the lifetime of an extra electron is longer than the transit time (τ_life_ > τ_tr_), the extra electron reaches the anode, and another electron immediately enters the photoconductor to maintain the charge neutrality and drifts to the anode terminal. This process is repeated until the extra electron recombines with a hole. This process takes τ_life_ on average, and the gain is greater than unity. However, if τ_life_ < τ_tr_, the extra electron recombines with a hole before the transit is completed. To achieve a gain of greater than unity without multiple electron–hole pair production, a higher power by an external circuit is needed. Equation (3) calculates the photocurrent of the photodetector^39^.

3 $$I_{ph} = \mu V_{DS} (C_{ox} \Delta V_{G} )\left( \frac{W}{L} \right),$$

where ${C}_{ox}$ is the dielectric capacitor per unit area, and W and L are respectively the width and length of the graphene channel.

From Eq. (4), the net photocurrent is independent of the thickness of the SiO_2_ film, while it depends on the carrier transit time and the amount of photoinduced electric charge as follows:

4 $$I_{ph} = \Delta Q/\tau_{tr} ,$$

where ∆Q is the amount of photoinduced electric charge, and τ_tr_ is the transit time of the carrier in the graphene channel.

As shown in Eq. (5), the amount of current generated in this type of detector depends on α and d as given below^40^.

5 $$I = I_{0} e^{ - \alpha d} ,$$

where $\alpha$ is the absorption coefficient, and d is the absorber layer thickness.

To increase the current, materials with a high absorption coefficient are used because according to Eq. (6), with increasing d, C_ox_ decreases. Moreover, according to Eqs. (7) and (8), the decrease in C_ox_ reduces g_m_, and as a result, photocurrent decreases. Therefore, the absorber layer thickness, d, should have an optimal limit, which is equal to 81 nm (Nb_2_O_5_) in the present study. The oxide-to-metal conversion factor of Nb is 2.7, meaning that for 1 nm of Nb, 2.7 nm Nb_2_O_5_ is formed.

6 $$C_{ox} = \varepsilon \varepsilon_{0} \frac{1}{d},$$

where $\varepsilon$ is the dielectric constant, and ${\varepsilon }_{0}$ is the vacuum permittivity.

7 $$g_{m} = \mu C_{ox} V_{DS} \left( \frac{W}{L} \right),$$

where ${g}_{m}$ is the transconductance given as follows:

8 $$g_{m} = \frac{{\partial I_{d} }}{{\partial V_{g} }},$$

where V_GS_ is the control voltage.

## Results and discussion

**Figure 4:**
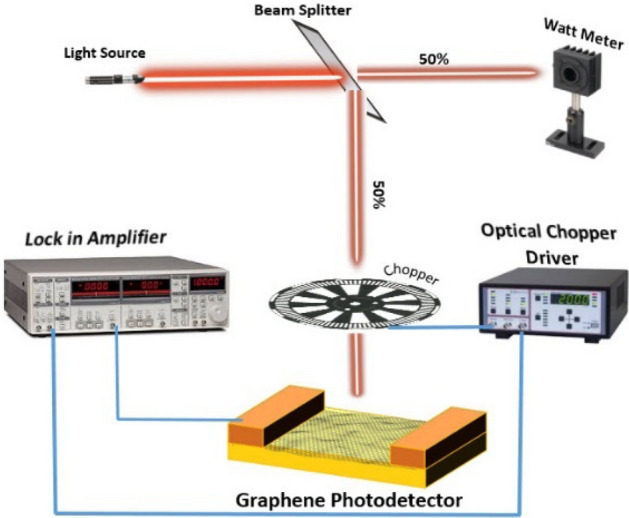
Optical characterization setup. The setup includes a laser, a beam splitter, a lock-in amplifier (Bentham), and an optical chopper.

Some of the figures of merit (FOMs) of the photodetectors are evaluated optically. The optical characteristics, except for some special cases, were obtained using a helium-cadmium laser at a power of 1 μW, a wavelength of 325 nm, and a chopper frequency of 3 kHz. As shown in Fig. 4, for optical characterization, the laser light hits a mirror, and the reflected light reaches a chopper and then the photodetector. The detector output is connected to the input of a lock-in amplifier.

Using this optical setup has two advantages. The first advantage is that the ambient noise is eliminated, and the second advantage is that even the lowest amount of photocurrent can be observed with this setup. The photoconductive and photogating photodetectors with Nb_2_O_5_ and TiO_2_ absorber layers were optically examined.

As shown in Table 1, the Nb layer was deposited on the silicon substrate by an electron beam device, and the Nb_2_O_5_ layer was obtained by anodizing with an oxide-to-metal conversion factor of 2.7. The Ti layer was deposited on the silicon substrate by sputtering, and TiO_2_ was obtained by the oxidation method with an oxide-to-metal conversion factor of 1.7.

**Table 1:** Deposition of the Nb_2_O_5_ and TiO_2_ absorber layers.

Absorber layer	Metal formation	Oxidation method	Oxide-to-metal ratio
Nb_2_O_5_	Ebeam	Anodization	2.7
TiO_2_	Sputtering	Thermal	1.7

**Figure 5:**
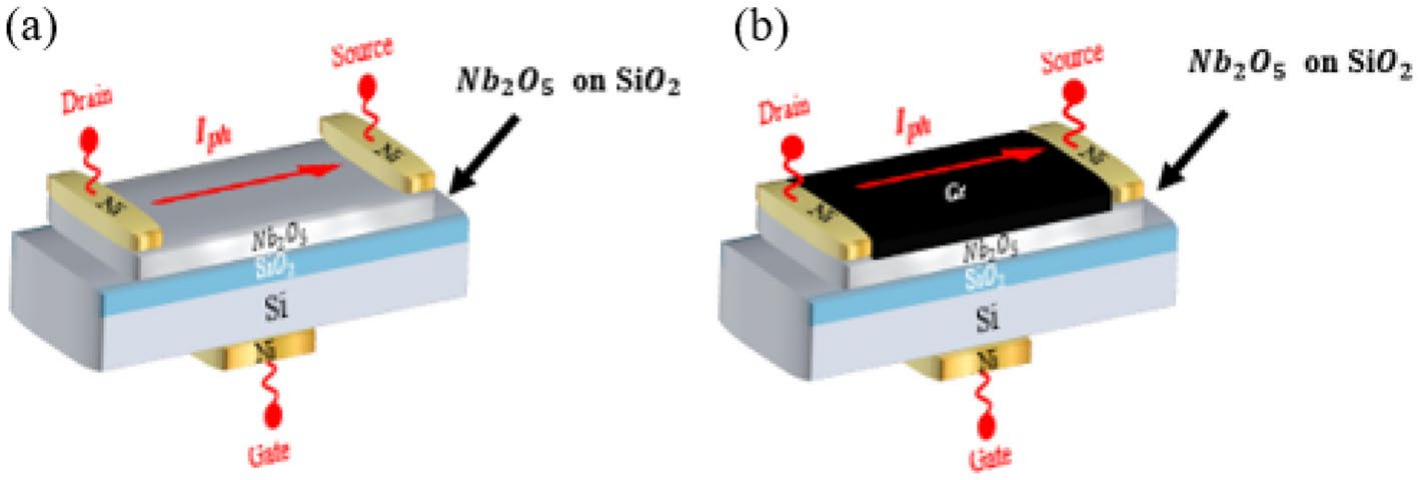
(**a**) Nb_2_O_5_ photoconductive photodetector profile and (**b**) Nb_2_O_5_ photogating photodetector profile and the points of connecting V_DS_ and V_GS_.

Figure 5a shows the profile of the Nb_2_O_5_ photoconductive detector, and Fig. 5b shows the profile of the Nb_2_O_5_ photogating detector. As shown in Fig. 5, the drain-source voltage (V_DS_) is applied to both ends of the graphene layer, and the control voltage (V_GS_) is applied to the silicon substrate through ohmic contacts.

**Figure 6:**
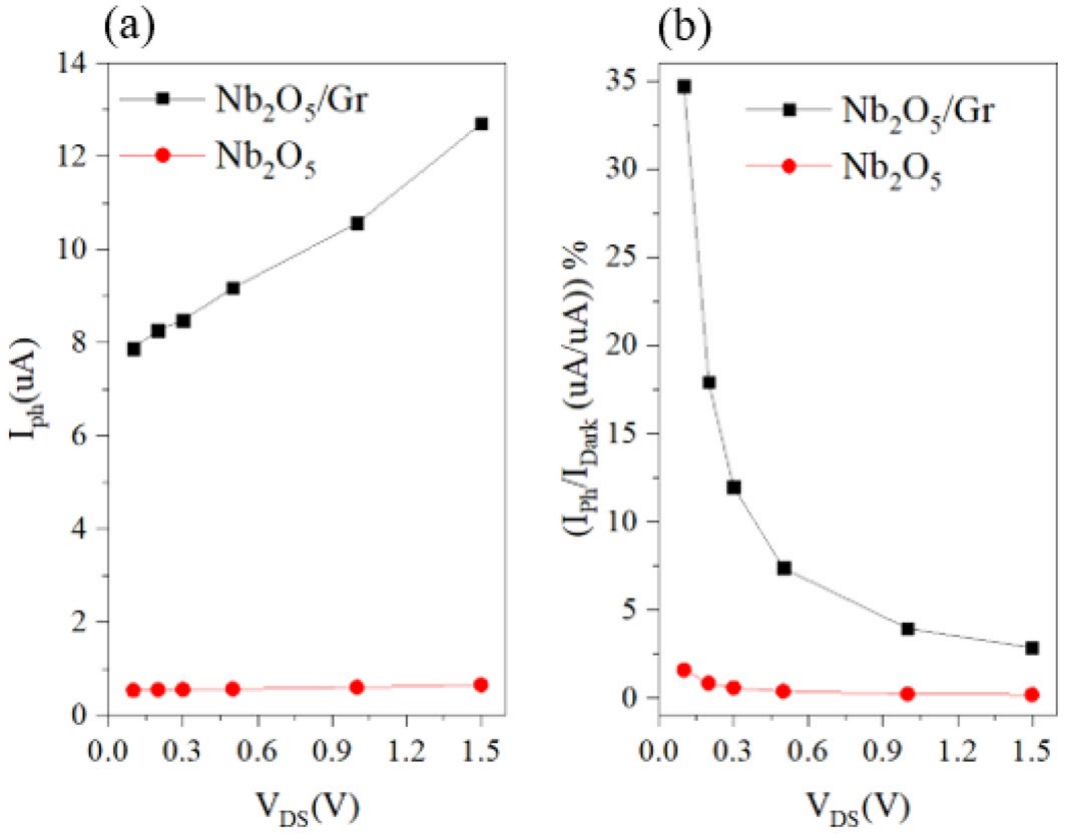
(**a**) Changes in photocurrent in microamps for the photoconductive and photogating detectors with Nb_2_O_5_ absorber layer versus drain-source voltage and (**b**) changes in the percentage ratio of photocurrent to dark current of the photoconductive and photogating detectors with Nb_2_O_5_ absorber layer versus drain-source voltage for P = 1 μW and V_GS_ = 1 V.

Figure 6a shows the photocurrent in μA versus drain-source voltage for the photoconductive and photogating photodetectors with the Nb_2_O_5_ absorber layer. The photocurrent of the photoconductive photodetector increases from 0.533 to 0.64 μA. By adding graphene, the photocurrent increases from 7.87 and 12.69 μA in the drain-source voltage range of 0.1–1.5 V. As the value of V_DS_ increases, due to the reduction in the potential barrier, electrons more easily travel from Nb_2_O_5_ to graphene, and as a result, the increase in I_ph_ is greater. In other words, according to Eq. (6), with the increase in V_DS_, g_m_ and as a result, I_ph_ increases. Figure 6b shows the percentage ratio of photocurrent to dark current for the photoconductive and photogating photodetectors with the Nb_2_O_5_ absorber layer. With increasing drain-source voltage, the ratio of photocurrent to dark current decreases, and the lowest value is observed at the drain-source voltage with the highest responsivity, i.e., at 1.5 V. Not only responsivity changes slightly with the increase in voltage, but also a further increase in V_DS_ allows much current to pass through the photodetector, and the device heats up. This has an adverse effect on the performance of the device. As shown in Fig. 6a, the value of I_ph_ has a direct relation with V_DS_, while I_dark_ depends not only on V_DS_ but also on other factors, such as carrier lifetime. Therefore, the trend of the I_ph_/I_dark_ curve results from the differences between the slopes of I_ph_ and I_dark_. In the photoconductive and photogating detectors, the percentage ratios of photocurrent to dark current are 0.16% and 2.84%, respectively, at a wavelength of 325 nm, a V_DS_ of 1.5 V, and a power of 1 μW.

**Figure 7:**
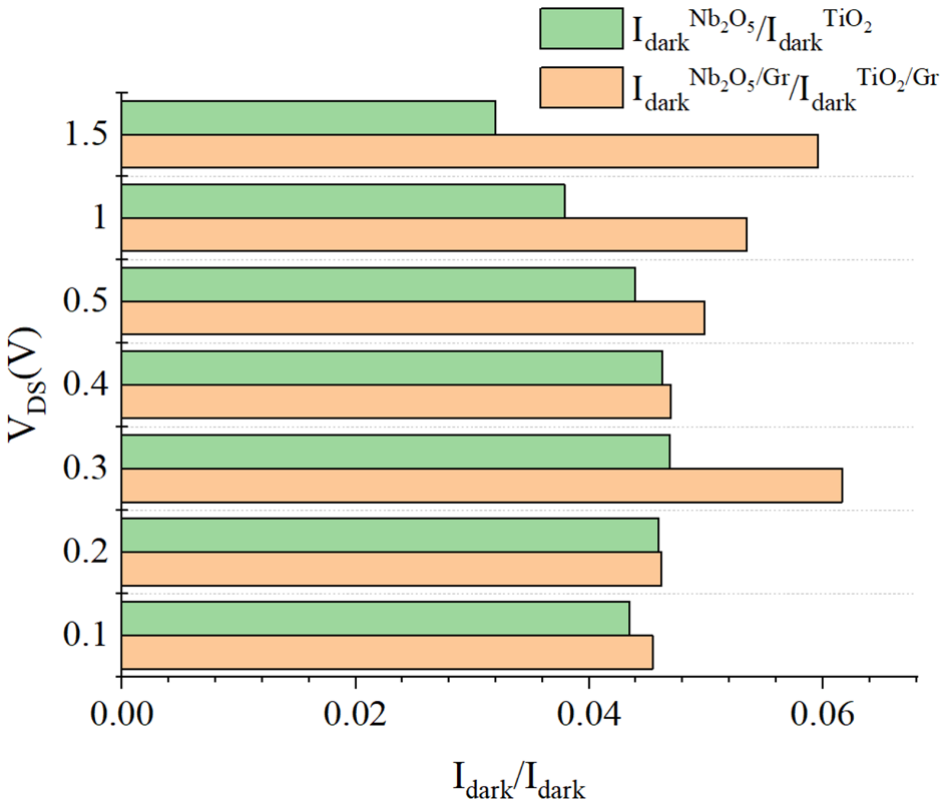
Ratio of the dark current of the photoconductive photodetector with Nb_2_O_5_ to that with TiO_2_ and the ratio of dark current of the photogating photodetector with Nb_2_O_5_ to that with TiO_2_, versus drain-source voltage for P = 1 μW and V_GS_ = 1 V.

A simple criterion is defined to compare the dark current of photoconductive and photogating detectors at different drain-source voltages. In Fig. 7, the ratios of the dark current of the detectors are compared. This figure shows the ratio of the dark current of the photoconductive photodetector with Nb_2_O_5_ to that with TiO_2_ and the ratio of dark current of the photogating photodetector with Nb_2_O_5_ to that with TiO_2_, versus drain-source voltage. In general, the ratio of the dark current with the Nb_2_O_5_ absorber layer to the dark current with the TiO_2_ absorber layer is higher in the photogating detector than in the photoconductive detector. The dark currents of the photogating and photoconductive detectors with the Nb_2_O_5_ layer are better than those of the photogating and photoconductive detectors with the TiO_2_ absorber layer.

**Figure 8:**
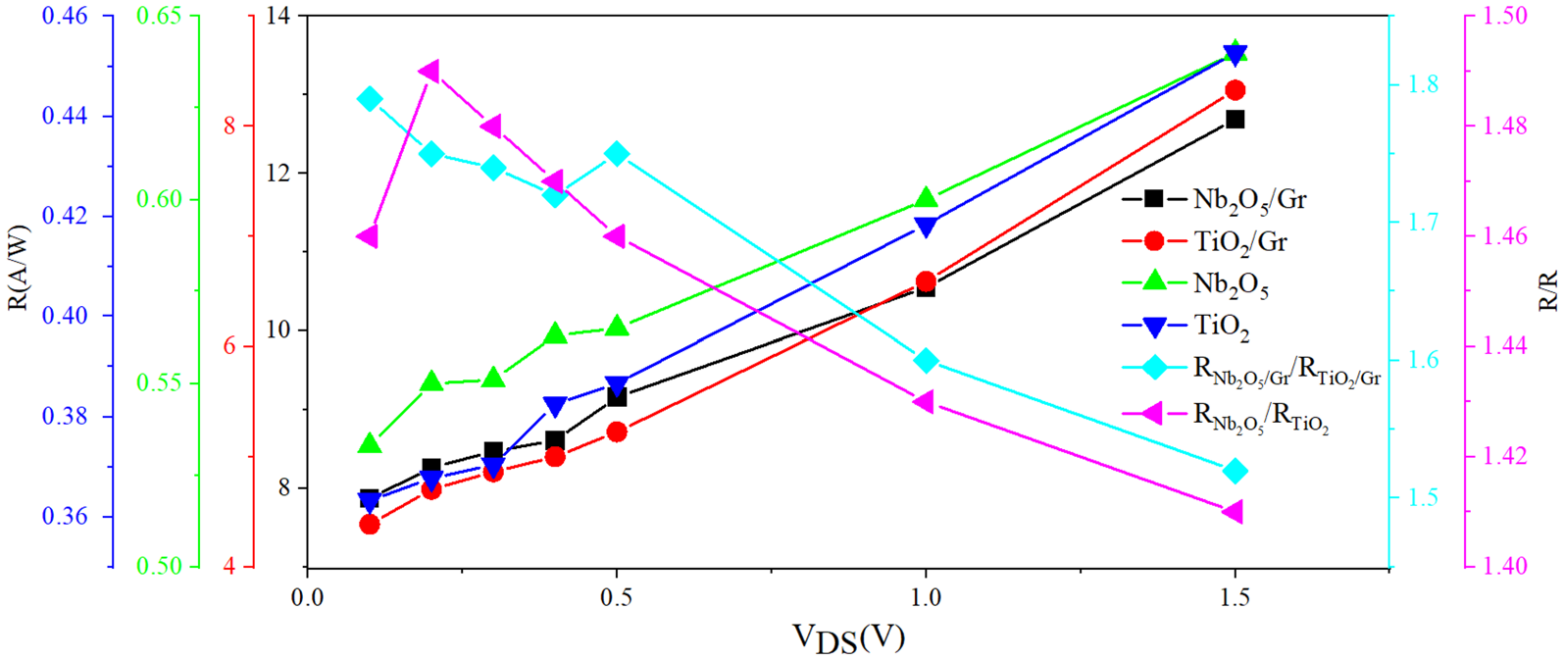
Responsivity of the photogating and photoconductive photodetectors (left axis). The ratio of the responsivity of the Nb_2_O_5_ photogating photodetector to that of the TiO_2_ photogating photodetector, and the ratio of the responsivity of the Nb_2_O_5_ photoconductive photodetector to that of the TiO_2_ photoconductive photodetector (right axis) at different drain-source voltages, P = 1 μW, and V_GS_ = 1 V.

Figure 8 shows the responsivities of four photoconductive and photogating photodetectors with Nb_2_O_5_ and TiO_2_ absorber layers in the V_DS_ range of 0.1–1.5 V. The photogating photodetector with the Nb_2_O_5_ absorber layer has the highest responsivity at all V_DS_ voltages. Moreover, the responsivities of the photoconductive detectors do not change significantly with increasing V_DS_ unlike the two photogating photodetectors. However, due to the photogating effect of the two detectors (i.e., detectors with Nb_2_O_5_/Gr and TiO_2_/Gr layers), the carrier trapping time increases, and thus, the responsivity and photocurrent increase. The responsivity is also dependent on the initial energy level of the graphene layer. At a drain-source voltage of 1.5 V, the ratio of responsivity of the Nb_2_O_5_/Gr photodetector to that of the TiO_2_/Gr photodetector is about 2, and the ratio of the responsivity of the Nb_2_O_5_ photodetector to that of the TiO_2_ photodetector is about 1.5. This indicates the better performance of the Nb_2_O_5_ absorber layer compared to the TiO_2_ absorber layer while both of these layers were made under the same laboratory conditions.

As shown in Table 2, the responsivities of the photogating detectors are better than those of the photoconductive detectors. The responsivities of both types of detectors with the Nb_2_O_5_ absorber layer are higher than those with the TiO_2_ absorber layer. The value of ${I}_{dark}^{{Nb}_{2}{O}_{5}/Gr}/{I}_{dark}^{Ti{O}_{2}/Gr}$ of the photogating detectors is larger than the value of ${I}_{dark}^{{Nb}_{2}{O}_{5}}/{I}_{dark}^{Ti{O}_{2}}$ of the photoconductive detector.

**Table 2:** Summary of some FOMs at three different V_DS_ values for the photoconductive and photogating samples with the Nb_2_O_5_ and TiO_2_ absorber layers.

V_DS_ (V)	0.1	0.5	1.5
R (A/W) (Nb_2_O_5_/Gr)	7.87	9.16	12.69
R (A/W) (Nb_2_O_5_)	0.533	0.565	0.65
R (A/W) (TiO_2_/Gr)	4.3863	5.227	8.329
R (A/W) (TiO_2_)	0.36325	0.3867	0.4527
$\frac{{I}_{dark}^{{Nb}_{2}{O}_{5}/Gr}}{{I}_{dark}^{Ti{O}_{2}/Gr}}$	0.0454	0.04988	0.5956
$\frac{{I}_{dark}^{{Nb}_{2}{O}_{5}}}{{I}_{dark}^{Ti{O}_{2}}}$	0.04346	0.04393	0.03195

**Figure 9:**
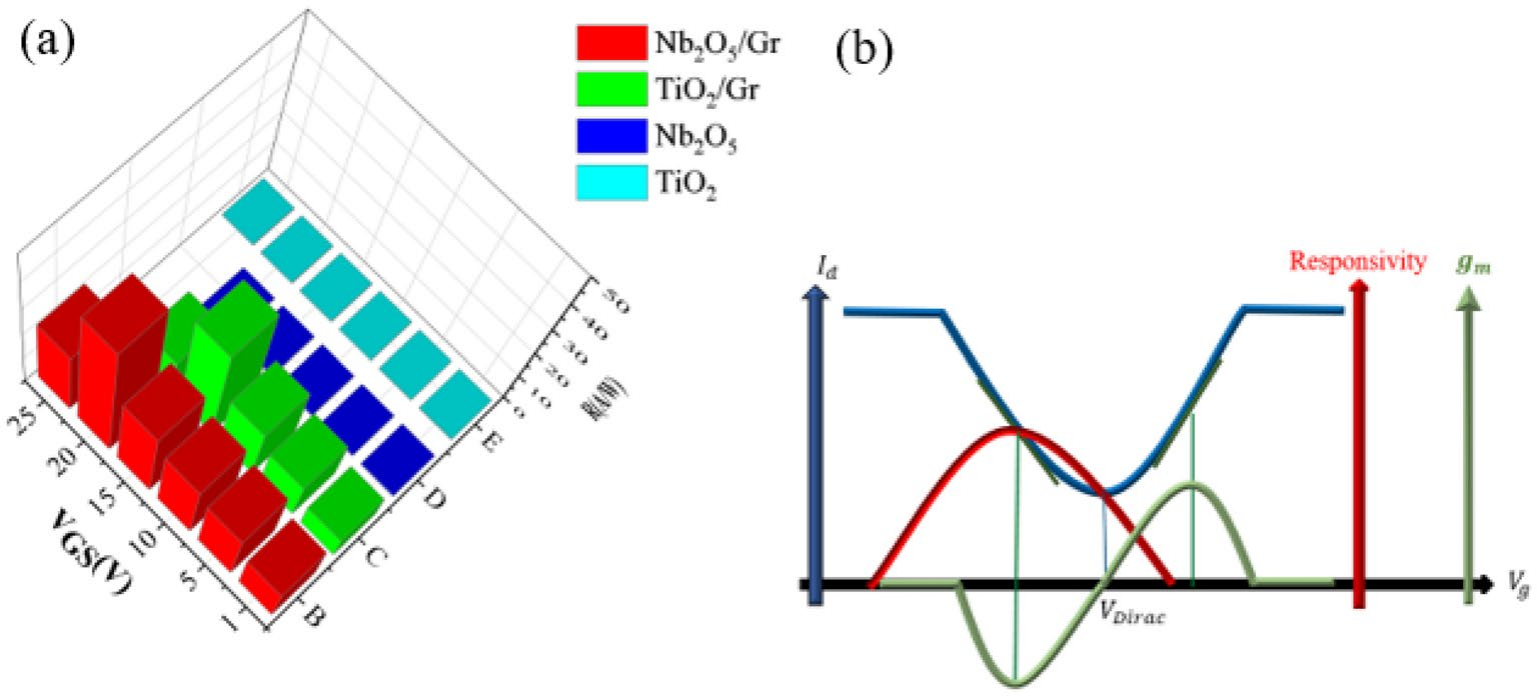
(**a**) Responsivity of the photoconductive and photogating detectors versus gate voltage at P = 1 μW and V_DS_ = 0.5 V, and (**b**) blue curve (Dirac point), red curve (responsivity), and green curve (gm = ∂I_d_/∂V_g_).

Figure 9a shows the responsivity in terms of gate voltage. As shown in this figure, the responsivities of the photoconductive detectors do not change significantly with the increase in V_GS_. However, in the photogating detectors, the carrier trapping time increases due to the photogating effect, so responsivity and photocurrent increase. The responsivity is also dependent on the initial energy level of the graphene layer. Therefore, it is proven that the increase in the responsivity of the photodetector with increasing V_GS_ is because of transferring the graphene layer to the TiO_2_ absorber layer.

As shown in Fig. 9a, with increasing gate voltage, the responsivity of the photogating detectors increases. The Dirac points of the Nb_2_O_5_ and TiO_2_ photogating detectors are located at 20 V and 15 V, respectively. At the Dirac points, not only the photogating detectors have the highest responsivity, but also the curves have the highest slope around the Dirac points. As shown in the curves of the photogating detectors, further increase in the gate voltage has a downward trend, and responsivity decreases. The reason for this is shown in Fig. 9b. In this figure, the blue curve shows the photocurrent in terms of the gate voltage in the graphene material. The minimum of the blue curve is the Dirac point. The Dirac voltage corresponds to when the numbers of electrons and holes are the same. As it is clear from the blue curve, at the gate voltages that are far away from the Dirac point, the photocurrent is independent of the gate voltage, so increasing or decreasing the gate voltage does not affect the value of photocurrent. As shown, the steepest slope of the curve is somewhere near the Dirac point. From Eq. (8) and the green curve, this slope is at gm = ∂I_d_/∂V_g_, and the maximum of the responsivity curve, which is shown in red, is located at the points with the highest slope.

**Figure 10:**
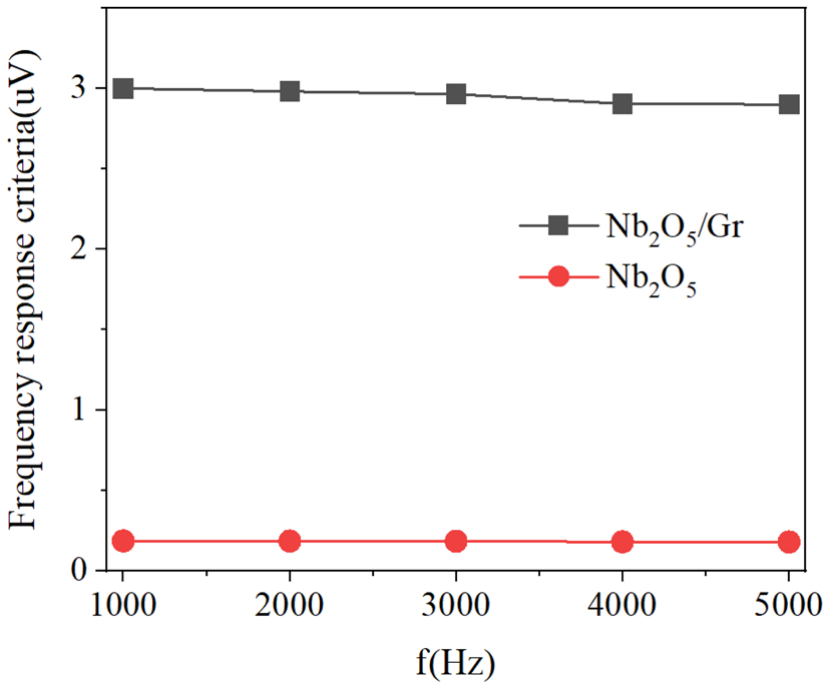
Frequency response criteria of the photoconductive and photogating detectors with Nb_2_O_5_ absorber layer at P = 1 μW, V_DS_ = 0.5 V, and V_GS_ = 1 V.

As shown in Fig. 10, the bandwidth of the photoconductive and photogating photodetectors with an Nb_2_O_5_ absorber layer was calculated as an FOM. With the increase in frequency, the frequency response criterion does not decrease substantially. These results indicate that both photodetectors can be used in applications, such as UV imaging and many modern systems, with a frequency requirement of up to 5 kHz.

Table 3 compares the photoconductive/photogating detectors with the Nb_2_O_5_/TiO_2_ absorber layers (presented in this study) and previous detectors presented in other articles with the same absorber layer materials. According to this table, detectors with the Nb_2_O_5_ absorber layer have better responsivity than those with the TiO_2_ absorber layer. The Nb_2_O_5_ Nanobelts and the Nb_2_O_5_ nanoplates detectors have a better responsivity than the thin-film Nb_2_O_5_ photoconductive and photogating detectors. The Nb_2_O_5_ nanoplates detector has a better responsivity than the Nb_2_O_5_ Nanobelt, but the fabrication of the thin-film Nb_2_O_5_ detector is simpler than Nb_2_O_5_ nanoplates and nanobelts detectors.

**Table 3:** Comparison of the responsivities of detectors with the Nb_2_O_5_ and TiO_2_ absorber layers presented in this study and previous articles.

UV photodetector	Year	Responsivity	References
Nb_2_O_5_ Nanbelts	2011	15.2 A/W at 320 nm	^31^
Nb_2_O_5_ nanoplates	2015	24.7 A/W at 320 nm	^32^
TiO_2_ nanotubes/gaphene	2016	0.126 A/W at 365 nm at 5 mW	^41^
TiO_2_/graphene	2018	80 mA/W at 1 V and 1 μW	^37^
Nb_2_O_5_	Present work	0.65 A/W at 1 V and 1 μW	–
TiO_2_	Present work	0.45 A/W at 1 V and 1 μW	–
Nb_2_O_5_/Gr	Present work	12.69 A/W at 1 V and 1 μW	–
TiO_2_/Gr	Present work	8.32 A/W at 1 V and 1 μW	–

## Conclusion

An Nb_2_O_5_ absorber layer was used for fabricating a graphene detector based on the photogating effect. Moreover, an anodizing process was employed to produce this absorber layer from a thin layer of Nb. These processes have not been conducted in the literature and were presented in this study for the first time. It is worth noting that compared to photodetectors with a TiO_2_ absorber layer, the photodetectors with an Nb_2_O_5_ absorber layer performed better in terms of the cost, simplicity of the fabrication process, and high value of responsivity. With 325-nm laser radiation, 1 μW power, at a drain-source voltage of 1.5 V, and a gate voltage of 1 V, the responsivities of the photoconductive and photogating detectors with the TiO_2_ absorber layer were 0.45 A/W and 8.32 A/W, respectively, while the responsivities of the photoconductive and photogating detectors with the Nb_2_O_5_ absorber layer were 0.65 A/W and 12.69 A/W, respectively. Moreover, the percentage ratios of the photocurrent to dark current of the photoconductive and photogating detectors with the TiO_2_ absorber layer were 0.003% and 0.111%, respectively. However, the percentage ratios of the photocurrent to dark current of the photoconductive and photogating detectors with the TiO_2_ absorber layer were 0.16% and 2.84%, respectively. Therefore, the responsivities and the percentage ratios of the photocurrent to dark current in detectors with the Nb_2_O_5_ absorber layer were better than the detectors with the TiO_2_ absorber layer.

## Data Availability

The datasets used and analyzed during the current study are available from the corresponding author upon reasonable request.
